# Cryoablation for the treatment of breast cancer: immunological implications and future perspectives. Utopia or reality?

**DOI:** 10.1007/s11547-024-01769-z

**Published:** 2024-01-31

**Authors:** Francesca Galati, Andrea Marra, Federica Cicciarelli, Marcella Pasculli, Roberto Maroncelli, Veronica Rizzo, Giuliana Moffa, Federica Pediconi

**Affiliations:** Department of Radiological, Oncological and Pathological Sciences, Sapienza University of Rome, Viale Regina Elena 324, 00161 Rome, Italy

**Keywords:** Cryoablation, Breast cancer, Ultrasound, Breast MRI, CEM

## Abstract

Cryoablation is a minimally invasive technique currently employed in breast cancer care, that uses freeze and thaw cycles to treat benign breast lesions, small breast cancers or focal sites of metastatic disease in patients not eligible for surgery. The final goal of this procedure is to destroy breast cancer cells using extreme cold. In addition, several studies have shown that this technique seems to have an enhancing effect on the immune response, especially by increasing the expression of tumor neoantigens specific to tumor cells, which are then attacked and destroyed. Exploiting this effect, cryoablation in combination with immunotherapy could be the key to treating early-stage breast cancers or patients who are unsuitable for surgery. According to some recent studies, there are other potential tools that could be used to enhance the therapeutic effect of cryoablation, such as FE3O4 nanoparticles or the manipulation of aquaporin expression. The aim of this narrative review is to summarize the current evidence regarding the use, indications, advantages and disadvantages of cryoablation in the treatment of breast cancer.

## Introduction

Breast cancer is the most diagnosed cancer in female population and is the fifth leading cause of cancer death worldwide, with an estimate of 2.3 million cases and 685,000 deaths in 2020 [1], with a projection of 4.4 million cases in 2070 [2]. Globally, breast cancer accounts for about 24.5% of all cancer cases, causing 15.5% of cancer deaths and ranking first in incidence and mortality in most countries of the world. Mammography screening allows cancers to be detected at an earlier stage, and, as a result, the wider use of mammography worldwide has led to an increase in breast cancer detection rate. Other causes for the increase in breast cancer diagnoses include an aging population, delayed marriage age, hormone replacement therapy, use of contraceptives, obesity, alcohol consumption, smoking, unbalanced diet, environment toxicant and no physical activity [3, 4]. In this context, breast-conserving treatments, such as local excision or quadrantectomy with associated radiotherapy and/or chemotherapy, are generally preferred in breast cancers up to 3 cm in size [5–7]. However, the consideration of histopathology and molecular subtype profiling are certainly essential elements in the choice of breast cancer treatment these days. In recent years, cryoablation, a minimally invasive technique, has become a viable alternative to surgery in the treatment of selected breast cancer patients who are not candidates for surgical resection [8], with the advantages of sidestepping the risks of surgical complications, reducing patient discomfort and achieving a better cosmetic outcome. The efficacy of cryoablation was highlighted in a 2017 systematic review by Mauri et al. [9]. In fact, out of 156 lesions treated with cryoablation, 95% success of the technique and 75% efficacy of the technique were reported. Specifically, technical success is defined as the rate of patients in which the operator was able to technically complete the ablation procedure, while technical efficacy is defined as the rate of lesions completely removed. These results seem to be related to the strengthening effect of cryoablation on the immune system, through the release of numerous cytokines and neoantigens, which stimulate the innate immune response. Furthermore, some studies have obtained very positive results from the association of cryoablation with an immunological therapy [10]. Therefore, in this view, the combination of cryoablation and immunotherapy could be the key to the immune treatment of neoplastic lesions [11, 12].

## Cryoablation

**Fig. 1:**
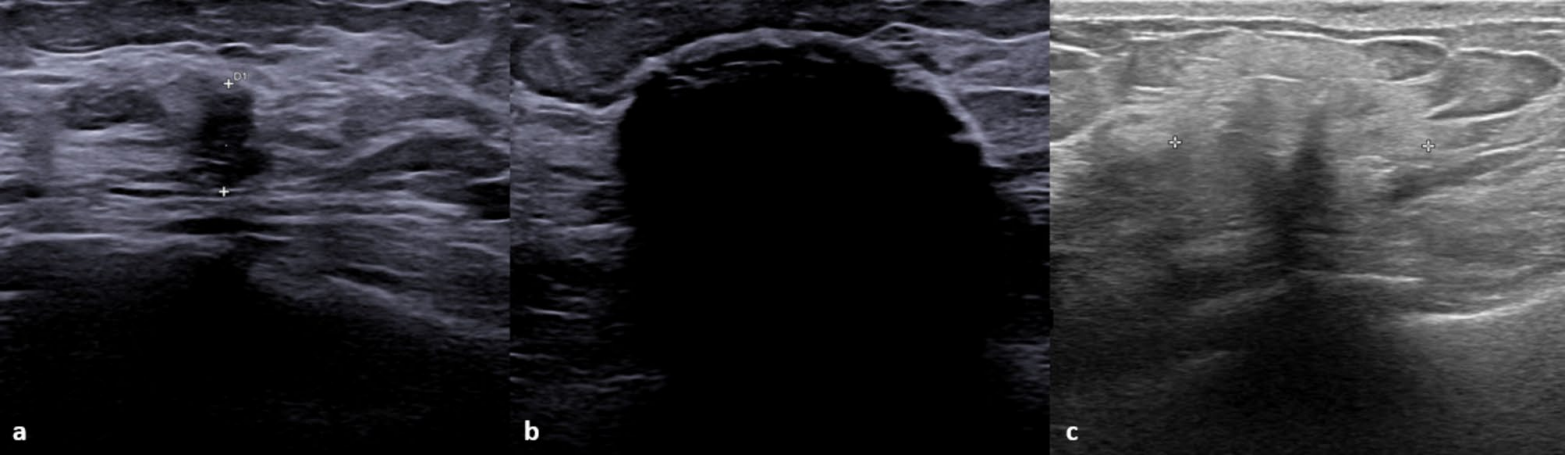
**a** Nine mm invasive breast carcinoma of no special type seen on US before cryoablation; **b** Iceball encompassing the lesion during the ultrasound-guided procedure; **c** Cryoablated area after 15 days

Cryoablation is a minimally invasive percutaneous technique that involves the use of extreme cold by inserting a cryoprobe into the target tissue and alternating two freeze–thaw cycles, with the ultimate goal of forming an ice ball that encompasses and ablates the target lesion [13]. Cryoablation tools include one or more disposable 17-gauge cryoprobes**,** a cryogen such as argon or liquid nitrogen, and a resistance heater. Breast cryoablation is usually performed under ultrasound guidance and, after administration of a local anesthetic, a small skin incision is made through which the cryoprobe is inserted (Fig. 1). The whole procedure takes about 25–30 min: the first freezing cycle (10 min), a thawing cycle (5–10 min) and the second freezing cycle (10 min).

In particular, during the first freezing cycle, in addition to the cell damage due to ice crystals formation, the freezing of extra- and intracellular water occurs, creating an osmotic gradient that causes water to leak out of cells, dehydrating them. In the first thawing cycle, however, the osmotic gradient is reversed and water rapidly enters the cells, destroying their cell membranes. This results in the release of tumor antigens that are recognized by the professional antigen presenting cells (APCs). The second freeze/thaw cycle has a larger area of effect because the tissue damaged during the first freeze cycle is able to conduct cold temperatures to the surrounding tissue more effectively. This causes coagulative necrosis mainly in the central area of the ablation zone, while more peripheral tissues are damaged mainly by delayed apoptosis mechanisms caused by mitochondrial damage [14]. Another indirect mechanism of damage is cellular ischemia, due to the destruction of microcirculation [15, 16]. The effects of these processes cause a local inflammatory response, followed by the release of tumor antigens and type 1 cytokines [17, 18], recruitment of APCs and tumor-specific T-cell response [19, 20]. Over time, the cryoablated tissue is resorbed and replaced by collagen streaks [14].

## Indications

According to the American Society of Breast Surgeons (ASBrS) guidelines, cryoablation has been approved for clinical use only for the treatment of fibroadenomatous lesions, while the use of cryoablation for breast cancer lesions is still in the experimental phase [21–23]. Current indications for cryoablation treatment of fibroadenomas are: lesion clearly visible on ultrasound, histologically confirmed diagnosis of fibroadenoma on core biopsy prior to treatment, and mass less than 4 cm in greatest diameter [21]. In two large follow-up studies, of approximately 40 fibroadenomas treated with cryoablation, a median reduction in mass volume of 99% was observed at 12 and 30 months ultrasound follow-up, respectively [24, 25]. As in the case of fibroadenomas, for the correct treatment plan of malignant lesions, it is essential to evaluate the location and size of the lump and the size of the affected breast. Tumors suitable for cryoablation are well defined lesions on breast ultrasound, located at least 5 mm and ideally more than 1 cm from the skin surface, the chest wall, the pectoral muscle and the nipple [26–28]. Ductal carcinoma in situ, invasive lobular carcinoma and invasive carcinoma with a predominantly intraductal component are not among the indications for cryoablation, because of the lack of ultrasound evidence of the lesion and because of the greater potential for extension beyond the ablation zone [29]. Less aggressive, unifocal and unilateral lesions are more favorable for cryoablation treatment, such as invasive ductal carcinoma positive for hormone receptor and negative for human epidermal growth factor receptor 2 (HER2) [29]. However, a number of studies are investigating the possibility of using this treatment in early, small and low-grade tumor lesions. In two large multicenter trials (FROST and ICE3), surgery was replaced by cryoablation procedure for the treatment of small breast tumors. Ultrasound-guided biopsy was performed about 6 months later to monitor for any residual focus of disease. The results were encouraging, with a 99% success rate [21, 30, 31]. According to the ASBrS guidelines, contraindications to cryoablation include the suspicion of a cystosarcoma phyllodes tumor or other malignancies, poor or no visualization of the lesion on ultrasound examination and a discordance between imaging and histopathological appearance of a fibroadenoma [21–23]. For patients with metastatic breast cancer (stage IV), the effectiveness of removing the primary tumor is still being debated. At present, salvage surgery is only indicated for locally advanced tumors with poor or no response to systemic drug treatment. However, there is growing interest in percutaneous ablation in stage IV patients, because it would significantly reduce the mass of the primary tumor without the complications of surgery. In a study by Pusceddu et al., 35 stage IV patients with mean tumor size of 3 cm underwent computed tomography-guided cryoablation, achieving complete tumor necrosis in 100% of cases at six months and, after 46 months, only 20% of patients developed local recurrences. This result underlines how cryoablation of the primary tumor in patients with metastatic breast cancer is a safe and effective technique, also considering the possibility of repeating the procedure in case of partial failure [32].

## Imaging follow-up

**Fig. 2:**
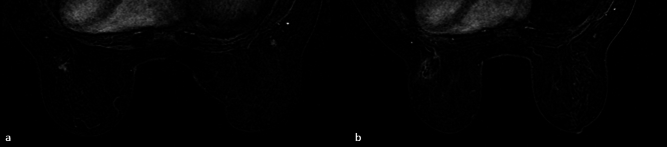
Axial T1-weighted post-contrast subtracted MR image shows **a** 12 mm invasive breast carcinoma in the left breast before cryoablation; **b** the cryoablated area after 11 days

After cryoablation, it is essential to start a follow-up process, to assess the success rate of the procedure. To do this, the imaging technique used should be as reliable as possible, in order to identify any residual disease and to perform a new cryoablation or surgery. Several imaging methods can be employed to evaluate cryoablation success, though the times and methods of post-treatment follow-up have not yet been clearly defined [30]. Immediately after the procedure, on ultrasound examination, the ablated area appears as a hypoechoic nodule surrounded by a hyperechoic halo. After about 6 months, the area assumes the typical characteristics of fat necrosis [29] and over time, the ablated mass becomes impossible to distinguish from the zone of mixed echogenic ablation. On the contrary, the role of mammography is more limited, although after a few months the ablated area takes the appearance of fat necrosis, with a white border that shrinks over time [29]. Magnetic resonance imaging (MRI) is probably the most effective modality in post-cryoablation follow-up, as the absence of enhancement in the ablation zone is the best imaging predictor of complete response to ablation [33]. Typical findings related to cryoablation are primarily lack of signal and the presence of a thin surrounding uniform border of enhancement [34] (Fig. 2).

However, MRI is not a routine part of the follow-up of fibroadenoma cryoablation and, if it is chosen, MRI is done at 6 months and then again at 1 and 2 years. The predictive value of MRI and its role in the follow-up of cryoablation has been discussed by several authors. According to a retrospective study by Poplack et al., MRI does not appear to be sufficiently accurate in predicting cryoablation outcomes [34]. On the other hand, in the study by Manenti et al., there is a good correlation between the MRI volume and the size of the histological specimens [35]. Finally, another study (Z1072) by Simmons et al., reported a negative predictive value of 81.2% for MRI [36], which significantly increases when MRI was associated with breast ultrasound and mammography. Finally, according to the study by Poplack et al., there is no particular correlation between the pathological findings seen on the excised specimens and those found on MRI. In fact, the gross and microscopic pathological analyses of the samples clearly show the presence of three pathologically distinct zones: a central red area, in which hemorrhage, ischemic alteration and coagulative necrosis are appreciated; a surrounding yellow ring, where acute and chronic inflammatory changes, fat necrosis and granulation tissue are observed; and a peripheral area where there is normal fat and fibroglandular tissue. However, there is no significant correlations between analysis of cryoablation parameters, MRI results, lesion and pathological characteristics [34]. Among the imaging techniques, to date the role of contrast-enhanced mammography (CEM) in cryoablation follow-up has not yet been highlighted. However, CEM has the potential to be a valid alternative, especially in those patients with contraindications to MRI, and may therefore be used more widely in the future.

## Advantages and disadvantages

Breast cryoablation has considerable advantages, it is a minimally invasive technique with a short duration, which does not cause pain to the patient during and after the treatment, it is an outpatient procedure that requires local anesthesia and ensures a favorable aesthetic result [36, 37]. The technique is also very well tolerated in cases of patients who are not candidates for or refuse surgery. Furthermore, according to a study conducted by Khan et al., cryoablation offers significant cost-effectiveness and quality of life advantages compared to surgery for early stage and low-risk breast cancers, also considering psychosocial and aesthetic factors [38]. According to some studies, long-term patient satisfaction is around 97% [24, 25]. The disadvantages of cryoablation are mainly technical and related to the high cost of argon gas and the size of the cylindrical container, which is also cumbersome to handle [39]. Side effects and complications include bleeding, frostbite, skin or chest wall injury, infection, local swelling, ecchymosis and incomplete or not possible treatment due to the location of the lesion to be treated. These are infrequent but possible events. [13, 21, 40].

## The role of immune system

In recent years, there is growing interest in the anti-tumor immune response induced by cryoablation. The cooling phase results in coagulative necrosis that mainly affects the central tissues in the ablation zone, while peripheral tissues undergo delayed apoptosis due to mitochondrial damage [16, 41]. During the thawing phase, tumor cells inside the ice ball release tumor antigens, nuclear proteins and proinflammatory cytokines. These signals attract macrophages, NK cells and granulocytes, stimulating the natural immune response and resulting in the release of APCs that reach the cryoablated tissue [16, 42]. According to many studies, the best way to enhance the immune response would be to block tumor checkpoints, allowing the immune system to recognize new cryoablated self-antigens. Thus, the combination of cryoablation and immunotherapy could be the key to immune treatment for neoplastic lesions. [11, 12]. Several studies have demonstrated the synergistic effects of this combined approach, such as a pilot study by McArthur et al. that combined cryoablation with Ipilimumab administration with encouraging results [10]. It has also been hypothesized that another particular effect, known as the "abscopal effect," occurs after cryoablation, which has also been studied in relation to radiotherapy. This effect results in a reduction of distant metastatic lesions [15], and the cause would be precisely the release of tumor-specific antigens that the immune system uses to trigger a specific response toward the tumor [43].

## New approaches to cryoablation

In recent years, many studies have investigated new methods to enhance the effects of cryoablation in treating cancer cells. A 2017 preclinical study by Ping Ye et al. [44] investigated the mechanisms and positive effects of Fe3O4 nanoparticles in cryoablative treatment of MCF-7 cancer cells. In particular, it was highlighted that Fe304 nanoparticles enhanced intracellular ice formation (IIF), the mechanism of cell apoptosis and recrystallization, showing that as the concentration of Fe3O4 increased, so did the ability of MCF-7 tumor cells to destroy it. Another study in 2021 [45] examined the role of aquaporin channels and the possible effects of their regulation in cryotherapy, which could be exploited in treatment. Water leakage, regulated by these channels, contributes to cancer cell damage during cryoablation. This preclinical study analyzed several aquaporins (AQP1, AQP3, AQP5) and their cellular arrangement after cryoablation in cultured breast cancer cells (MCF-7 and MDA-MB-231). Water leakage, regulated by these channels, contributes to cancer cell damage during cryoablation. It was found that tumor cells redistribute aquaporin proteins from the cytosol to the cell membrane, particularly AQP1. There is evidence that these water channels are associated with increased cell proliferation and invasion and are therefore upregulated in breast cancer cells, making AQP1 a potential prognostic marker for breast cancer [46, 47]. While it was previously believed that AQP1 induction occurs through estrogen stimulation [48], in this study it was observed that the increase in AQP1 expression after cryoablation occurred in MDA-MB-231 cells, which lack ER receptors, and not in MCF-7 cells, which possess the estrogen receptor [49]. This would demonstrate that the mechanism of aquaporin induction is independent of estrogen receptors. In light of this, understanding the real role of AQP 1 and its inhibition could play a key role in increasing cell damage during cryoablation and reducing the therapy failure rate in breast cancer treatment.

## Conclusions / State of the art and future perspectives

Several studies have shown that cryoablation is a useful minimally invasive technique for patients with breast cancer, since it is a well-tolerated procedure that achieves complete tumor ablation in a high percentage of cases. Overall, data from the literature are conflicting, as highlighted by a recent systematic review by Lanza et al., which reported variable local tumor control ranging from 19 to 95% [50]. Cryoablation may be a valuable technique for local treatment of patients who are unfit or refuse surgery and as an alternative to surgery in patients with early breast cancer. The latter is the most interesting issue because it could represent a conceptual shift toward minimally invasive treatment. Despite encouraging results, to date no study has shown cryoablation to be equal to breast-conserving surgery in terms of local control, disease-free survival or overall survival.

The effects of cryoablation on the immune system and possible synergistic effects with systemic therapies are open and appealing fields that deserve additional attention. Indeed, immunotherapy has recently emerged as a worthwhile treatment for several solid tumors. Currently, immune strategies include the use of drugs that modulate key T-cell checkpoint inhibitors. In particular, checkpoint inhibition along with other treatments, such as systemic therapies and local therapy, including cryoablation, seems to be a promising strategy in the treatment of breast cancer.

At present, surgery remains the standard local treatment of breast cancer, with radiation therapy if needed clinically. The value of cryoablation compared with traditional open surgery needs to be confirmed by large prospective studies. In the future, combination treatment schemes, including cryoablation and adjuvant procedures, as well as the growing experience in cryoablation of breast cancers, may potentially reduce the need for open surgery and reveal the full potential of cryotherapy application in breast cancers.
